# Effects of Partial Replacement of Palm Oil with *Schizochytrium* Oil on Rumen Microbiota, Ruminal Volatile Fatty Acid Concentrations, Rumen Epithelial Transcriptome, and Serum Metabolome in *Hu* Lambs Fed a Medium-Fiber Diet

**DOI:** 10.3390/microorganisms14102268

**Published:** 2026-10-06

**Authors:** Rongyu Yao, Ziang Wang, Xiuxiu Weng, Xiangpeng Yue, Wanhong Li

**Affiliations:** State Key Laboratory of Herbage Improvement and Grassland Agro-Ecosystems, Key Laboratory of Grassland Livestock Industry Innovation, Ministry of Agriculture and Rural Affairs, College of Pastoral Agriculture Science and Technology, Lanzhou University, Lanzhou 730020, China; 120220900691@lzu.edu.cn (R.Y.); wangza2024@lzu.edu.cn (Z.W.); wengxx@lzu.edu.cn (X.W.); lexp@lzu.edu.cn (X.Y.)

**Keywords:** *Schizochytrium* oil, docosahexaenoic acid, rumen microbiota, rumen fermentation, transcriptomics, serum metabolomics

## Abstract

*Schizochytrium* oil (SO) is rich in docosahexaenoic acid (DHA), but its ruminal effects in sheep remain unclear. Twenty *Hu* lambs were fed CK or SO diets for 84 d. The CK diet contained 4% palm oil, 31.43% neutral detergent fiber (NDF), and 5.58% ether extract (EE). The SO diet contained 3% palm oil and 1% SO, with similar NDF (31.76%) and EE (5.45%). Blood samples were collected one day before slaughter, before the morning feed replenishment, whereas rumen contents and epithelial tissue were collected after slaughter following a 16-h fast. Rumen microbiota, volatile fatty acids (VFAs), epithelial morphology (*n* = 10/group), RNA-seq (*n* = 4/group), and serum metabolomics (*n* = 5/group) were analyzed. The results showed that the rumen microbial community structure did not differ between the two groups (PERMANOVA, R^2^ = 0.101, *p* > 0.05), but SO selectively altered the relative abundance of seven genera (FDR < 0.05), including higher *Fibrobacter* and lower *Methanosphaera*. These changes were accompanied by higher acetate, propionate, butyrate, valerate, and total VFA concentrations and a lower acetate/propionate ratio (*p* < 0.05). Histological analysis showed that rumen papilla length and total epithelial thickness were higher in the SO group than in the CK group (2286.49 vs. 1764.30 μm and 114.52 vs. 80.07 μm, respectively; *p* < 0.05). Rumen epithelial RNA-seq showed enrichment of pathways related to VFA metabolism, energy metabolism, and fatty acid metabolism in the SO group (FDR < 0.01). Serum metabolomics showed higher levels of DHA and several DHA-containing lipids in the SO group (FDR < 0.05). Overall, partial replacement of palm oil with 1% SO selectively altered selected rumen genera and increased ruminal propionate concentration, accompanied by changes in rumen epithelial morphology and metabolism and increased serum DHA-related lipids. These findings support the potential of SO as a dietary DHA source for *Hu* lambs and provide evidence for its effects on the rumen and serum DHA-related lipid profile.

## 1. Introduction

Docosahexaenoic acid (DHA) is an important component of the fatty acid profile of lamb. Increasing DHA deposition in muscle can improve the nutritional value of meat, and DHA-rich dietary supplementation has also been shown to improve tenderness and flavor related traits [1,2]. Consumers have also shown greater purchase intention for meat products enriched with DHA [3]. However, DHA deposition in lamb is generally low, and the conversion of precursor fatty acids to DHA in sheep is limited [4]. Therefore, direct supplementation with DHA-rich lipid sources is an effective way to increase DHA deposition in lamb. Fish oil is a conventional source of DHA. However, animal-derived ingredients other than milk and dairy products are prohibited in ruminant feeds in China [5]. *Schizochytrium* is a marine heterotrophic protist and an important microbial source of DHA, which can account for more than 40% of total fatty acids in some strains [6]. Previous studies have shown that *Schizochytrium* supplementation increases DHA deposition in lamb muscle without affecting growth performance [7]. Thus, *Schizochytrium* supplementation may be a useful dietary strategy for increasing DHA in lamb.

In ruminants, dietary DHA undergoes lipolysis and biohydrogenation in the rumen before it is absorbed and deposited in meat and milk. *Schizochytrium* powder has been shown to affect specific rumen bacterial groups in sheep, including *Bacteroidales* related bacteria and *Ruminococcaceae* [8,9]. However, the form of DHA supplementation can also affect its metabolism and effects in the rumen. Compared with *Schizochytrium* powders, SO caused greater changes in the rumen microbiota [10]. For example, SO increased the relative abundances of *Ruminobacter* and *Succiniclasticum*, whereas *Schizochytrium* powders did not show the same changes [10]. Lipids in *Schizochytrium* biomass or powder are contained within a cellular matrix, whereas SO is the extracted lipid fraction. The loss of the cellular matrix during oil extraction may allow DHA to interact more directly with rumen microorganisms [11]. In addition, even at the same DHA supplementation level, *Schizochytrium* and SO produced different ruminal fatty acid and microbial responses [11]. Therefore, results obtained with *Schizochytrium* biomass or powder may not fully reflect the effects of SO in the rumen. Changes in the rumen microbiota also affect volatile fatty acid (VFA) concentrations. VFAs are major products of microbial fermentation and play important roles in rumen epithelial development and metabolism [12]. For example, VFAs such as butyrate and propionate are associated with rumen epithelial growth and the expression of genes related to cell proliferation and VFA transport [12,13]. However, studies on the effects of SO on rumen microbiota, VFA concentrations, and epithelial responses in sheep remain limited. It remains unclear whether SO feeding alters specific rumen microbial groups and VFA concentrations in sheep and whether these differences are associated with epithelial transcriptional responses.

Therefore, this study aimed to investigate the effects of dietary SO supplementation on rumen microbiota, VFA concentrations, and epithelial responses in *Hu* lambs, as well as changes in the serum metabolome. We therefore hypothesized that SO feeding would alter selected rumen microbial groups and VFA concentrations and that these changes would be associated with epithelial transcriptional responses. 16S rRNA gene sequencing, VFA analysis, rumen epithelial histology and transcriptomics, and untargeted serum metabolomics were used. This study may provide a better understanding of the ruminal and metabolic responses to dietary SO supplementation in sheep.

## 2. Materials and Methods

### 2.1. Animals, Diets, and Management

The animal experiment was conducted at Minqin Defu Sheep Industry Co., Ltd. (Minqin, Gansu Province, China). All animal procedures were reviewed and approved by the Animal Ethics and Welfare Committee of the Institute of Ruminant Research, Lanzhou University (approval no. CY20240712). A total of 20 healthy 3-month-old male *Hu* lambs were selected. The experiment consisted of a 7-day adaptation period followed by an 84-day experimental period. At the end of the adaptation period, all lambs were weighed individually, with an average initial body weight of 21.84 ± 0.47 kg. Lambs with similar initial body weights were then randomly assigned to two groups with 10 lambs per group. Body weight was recorded on days 0 and 84 after a 16-h fast, with free access to water. Overall average daily gain (ADG) was calculated as the difference between final and initial body weight divided by the 84-day experimental period.

The 10 lambs in each treatment were housed together in one outdoor pen with an earthen floor. Each pen was equipped with a long feed trough that provided sufficient space for all lambs to feed simultaneously and a water trough connected to an automatic water supply system. A mineral lick block was also provided in each pen. Lambs had continuous ad libitum access to feed throughout the experimental period. Fresh feed was replenished daily at 09:00 and 17:00 to ensure a continuous feed supply. Lambs had free access to clean water throughout the experiment. The mean ambient temperature and relative humidity during the experimental period were 20.6 °C and 38.8%, respectively. All lambs were treated for internal and external parasites and vaccinated according to the farm’s routine health management program. The vaccines included a goat pox live vaccine (TECON Pharmaceutical Co., Ltd., Suzhou, China) for the prevention of sheep pox and an inactivated combined vaccine against braxy, struck, lamb dysentery, and enterotoxemia (Jinyu Baoling Bio-Pharmaceutical Co., Ltd., Hohhot, China). Before the experiment, the pens were thoroughly cleaned and disinfected. During the experimental period, the pens were cleaned and disinfected once a week.

The experimental diets were formulated according to the National Research Council (NRC, 2007) and prepared as total mixed pelleted diets. The control (CK) group received the basal diet, whereas the SO group received the same diet in which 1% palm oil was replaced with an equal amount of SO. Each experimental diet was prepared in a single batch. The SO was purchased from Shandong Naichen Biotechnology Co., Ltd. (Jinan, China). According to the analytical report from Pony Testing International Group (Qingdao, China), DHA, EPA, EPA plus DHA, total n-3 fatty acids, and total n-6 fatty acids accounted for 49.7%, 0.558%, 50.3%, 51.0%, and 0.372% of total fatty acids, respectively. Based on feed intake records and dietary DHA content, the estimated average DHA intake was 2.24 g/lamb/day in the CK group and 6.16 g/lamb/day in the SO group. When normalized to the mean body weight during the experimental period, the corresponding DHA intakes were 62.1 and 168.5 mg/kg BW/day, respectively. Feed samples were collected from each diet before the morning feed replenishment on five consecutive days. Samples collected from the same diet were combined to form one composite sample for chemical analysis. The samples were dried at 65 °C for 48 h and ground through a 1-mm sieve. Dry matter (DM), crude protein (CP), ether extract (EE), ash, neutral detergent fiber (NDF), and acid detergent fiber (ADF) were determined according to AOAC (2012). Fatty acid determination followed protocols described in previous studies [14]. The ingredient and nutrient composition of the experimental diets are shown in Table 1. The fatty acid composition of the CK and SO diets were shown in Table 2. DHA accounted for 2.18% and 6.20% of total fatty acids in the CK and SO diets, respectively.

### 2.2. Sample Collection

Blood samples were collected one day before slaughter, before the morning feed replenishment. Each lamb was manually restrained by an assistant, and approximately 4 mL of blood was collected from the jugular vein by the same experienced operator into a vacuum tube without anticoagulant. The blood samples were allowed to clot at 25 °C for 1 h and then centrifuged at 3000× *g* for 15 min. The serum was immediately frozen in liquid nitrogen and then transferred to −80 °C for storage until subsequent analysis. Feed was withdrawn 16 h before slaughter.

The slaughter facility was located near the feeding facility. Before slaughter, all lambs were transferred to holding pens at the slaughter facility and kept there overnight until slaughter. The lambs were slaughtered by trained personnel according to the standard operating procedures of the facility.

Rumen contents were collected within 15 min after slaughter. A portion was immediately frozen in liquid nitrogen for 16S rRNA gene sequencing. Rumen fluid was obtained by filtering the contents through four layers of sterile gauze, immediately frozen in liquid nitrogen, and then transferred to −80 °C for storage until VFA analysis.

Rumen epithelial tissue was collected from the ventral sac of the rumen and gently rinsed with pre-cooled physiological saline. The tissue was divided into two portions. One portion was fixed in 4% universal tissue fixative (Sevier Biotechnology Co., Ltd., Wuhan, China) for 48 h for histological analysis, whereas the other was immediately frozen in liquid nitrogen and stored at -80 °C for transcriptomic analysis.

All 20 lambs were used for rumen microbiota, VFA, and rumen histological analyses. Four animals per group were randomly selected for RNA sequencing, and serum metabolomics was performed in five animals per group, including the four animals used for RNA sequencing.

### 2.3. Rumen Epithelial Histology

After fixation, the tissues were dehydrated through a graded ethanol series, cleared in xylene, embedded in paraffin, and cut into 5 μm sections. The sections were then deparaffinized, rehydrated, and stained with hematoxylin and eosin (HE). After staining, the sections were dehydrated, cleared in xylene, and mounted for microscopic observation. Images were acquired using an Olympus VS120 slide scanner (Olympus, Tokyo, Japan). Rumen epithelial morphology was measured using cellSens software (version 4.3; Olympus). Papilla length, papilla width, total epithelial thickness, and stratum corneum thickness were measured. Total epithelial thickness and stratum corneum thickness were measured in the non-papillary mucosal region, and the boundaries of the epithelial layers were defined according to Shao et al. [15]. For each animal, 3–6 intact papillae were measured for papilla length and width. The same number of measurements were obtained for total epithelial thickness and stratum corneum thickness. The investigator performing the measurements was blinded to group allocation. Measurements were averaged within each animal, and the animal mean was used for statistical analysis.

### 2.4. Rumen VFA Analysis

Rumen fluid samples were used for VFA analysis. Frozen rumen fluid samples were thawed at 4 °C and centrifuged at 13,500× *g* for 10 min. The supernatant (1.5 mL) was collected and mixed thoroughly with 0.2 mL of 1% (*w*/*v*) crotonic acid as an internal standard. The mixture was then passed through a 0.45 μm organic membrane filter before gas chromatographic analysis. VFA concentrations were determined using a TRACE 1300 gas chromatograph (Thermo Scientific, Milan, Italy) equipped with a DB-FFAP capillary column (30 m × 0.32 mm × 0.25 μm; Agilent Technologies, Santa Clara, CA, USA). The injector and detector temperatures were both set at 240 °C. The injection volume was 1 μL, and the split ratio was 50:1. The oven temperature was initially set at 50 °C, increased to 190 °C at 25 °C/min and held for 2 min, then increased to 200 °C at 10 °C/min and held for 5 min, and finally increased to 220 °C at 10 °C/min and held for 5 min. Air and hydrogen flow rates were 350 and 35 mL/min, respectively. Nitrogen was used as the carrier gas at a flow rate of 20 mL/min. Acetate, propionate, isobutyrate, butyrate, isovalerate, and valerate were identified by comparison with the retention times of the corresponding standards and quantified using the internal standard method. Total VFA concentration was calculated as the sum of the individual VFA concentrations, and the acetate/propionate ratio was calculated from the corresponding concentrations.

### 2.5. Rumen Microbiota Analysis

Rumen content samples were used for 16S rRNA gene sequencing at Novogene Biotechnology Co., Ltd. (Beijing, China). Microbial DNA was extracted using a modified cetyltrimethylammonium bromide (CTAB) method. After lysozyme treatment, DNA was purified by phenol–chloroform–isoamyl alcohol extraction, isopropanol precipitation, 75% ethanol washing, and RNase A treatment. The V4 region of the 16S rRNA gene was amplified using primers 515F (5′-GTGYCAGCMGCCGCGGTAA-3′) and 806R (5′-GGACTACNVGGGTWTCTAAT-3′). Each PCR reaction contained 15 μL of Phusion High-Fidelity PCR Master Mix (New England Biolabs, Ipswich, MA, USA), 0.2 μM of each primer, and approximately 10 ng of genomic DNA. PCR amplification was performed with an initial denaturation at 98 °C for 1 min, followed by 30 cycles of 98 °C for 10 s, 50 °C for 30 s, and 72 °C for 30 s, and a final extension at 72 °C for 5 min. PCR products were purified using magnetic beads. Sequencing libraries were quantified using a Qubit 2.0 fluorometer (Thermo Fisher Scientific, Waltham, MA, USA) and sequenced on an Illumina NovaSeq 6000 platform (Illumina, San Diego, CA, USA) using 250-bp paired-end reads. After removal of barcode and primer sequences, paired-end reads were merged using FLASH (version 1.2.11). Quality filtering was performed using fastp (version 0.23.1), and chimeric sequences were removed using vsearch (version 2.16.0). Sequence denoising was performed using DADA2 in QIIME 2 (version 2022.2) to generate amplicon sequence variants (ASVs). Taxonomic classification was performed against the SILVA 138.1 database. For alpha-diversity analysis, the ASV table was rarefied to the minimum sequencing depth across all samples before calculation of the Chao1 and Shannon indices. Taxonomic relative abundances were calculated from the unrarefied ASV count table as the count of each taxon divided by the total sequence count of the corresponding sample. Sequencing coverage was assessed using rarefaction curves and Good’s coverage.

Before beta-diversity and differential-abundance analyses, genera were retained when their mean relative abundance was >0.1% in either group. This filtering retained 63 of 283 genus-level categories. Bray–Curtis distances were calculated from the filtered relative-abundance matrix without renormalizing the retained values. Principal coordinate analysis (PCoA) and non-metric multidimensional scaling (NMDS) were used for visualization. Differences in microbial community structure between the CK and SO groups were tested by permutational multivariate analysis of variance (PERMANOVA) using the adonis2 function. Homogeneity of multivariate dispersion was assessed using PERMDISP. These analyses were conducted in R (version 4.4.1) using the vegan package (version 2.7.5). Microbial composition at the phylum and genus levels was expressed as relative abundance. Differences between the CK and SO groups were assessed using the Wilcoxon rank-sum test. Genera with FDR < 0.05 were defined as differentially abundant genera [16].

### 2.6. RNA Extraction, Library Construction, and Sequencing

Total RNA was extracted using TRIzol reagent (TIANGEN Biotech, Beijing, China) according to the manufacturer’s instructions. RNA concentration and purity were measured using a NanoDrop 2000 spectrophotometer (Thermo Fisher Scientific, Waltham, MA, USA). RNA integrity was assessed using an Agilent 2100 Bioanalyzer (Agilent Technologies, Santa Clara, CA, USA). Samples with at least 1 μg of total RNA, an RNA concentration of at least 100 ng/μL, an A260/A280 ratio of 1.8 to 2.0, and an RNA integrity number of at least 7 were used for library preparation. RNA libraries were prepared using the VAHTS Universal V10 RNA-seq Library Prep Kit (Premixed Version, Vazyme, Nanjing, China). Poly(A)+ mRNA was enriched using oligo(dT) magnetic beads and fragmented into short fragments. First-strand cDNA was synthesized using random hexamer primers, followed by second-strand cDNA synthesis. The cDNA was then subjected to end repair, dA-tailing, adapter ligation, fragment size selection, and PCR amplification. Library quality was assessed using an Agilent 2100 Bioanalyzer. Qualified libraries were sequenced by OE Biotech Co., Ltd. (Shanghai, China) on an Illumina NovaSeq 6000 platform (Illumina, San Diego, CA, USA) to generate 150 bp paired-end reads.

Raw reads were processed using fastp (version 0.20.1) to remove adapter sequences and low-quality reads. The Q30 and GC contents of the clean reads were calculated. Clean reads were aligned to the sheep reference genome ARS-UI_Ramb_v3.0 (GCF_016772045.2) using HISAT2 (version 2.1.0). Gene-level raw counts were obtained using HTSeq-count (version 0.11.2), and FPKM values were calculated for expression reporting. Principal component analysis (PCA) was performed using variance-stabilized count data. Differential expression between the CK and SO groups was analyzed using DESeq2 (version 1.22.2), with raw integer gene counts used as input. FPKM values were not used for differential-expression testing. Genes with FDR < 0.05 and |log2 fold change| ≥ 1 were defined as differentially expressed genes (DEGs). Kyoto Encyclopedia of Genes and Genomes (KEGG) pathway enrichment analysis was performed for Ovis aries using a hypergeometric test implemented with the phyper() function in R (version 4.4.1), with KEGG-annotated expressed genes as the background and Benjamini–Hochberg FDR correction. GSEA was performed using fgseaMultilevel in the fgsea package (version 1.30.0), with genes ranked by the DESeq2 Wald statistic and KEGG gene sets containing 10–500 genes.

### 2.7. Metabolite Determination

Serum samples were thawed on ice, vortexed for 10 s, and centrifuged at 12,000× *g* for 1 min at 4 °C. For metabolite extraction, 20 μL of serum was mixed with 20 μL of internal standard solution and 110 μL of methanol pre-cooled at −80 °C. The samples were vortexed for 1 min and incubated at −20 °C for at least 30 min. After centrifugation at 14,000× *g* for 10 min at 4 °C, the supernatant was vacuum freeze-dried and reconstituted in 100 μL of 10% methanol/90% water. The samples were vortexed for 30 s, sonicated for 1 min, and centrifuged again at 14,000× *g* for 10 min at 4 °C. Pooled quality control (PQC) samples were prepared by mixing 10 μL from each serum sample, and extraction solvent was used as the blank. Metabolomic analysis was performed using a Vanquish Flex UHPLC system (Thermo Fisher Scientific, Waltham, MA, USA) coupled to a Q Exactive HF-X mass spectrometer (Thermo Fisher Scientific, Waltham, MA, USA) equipped with a heated electrospray ionization source. Metabolites were separated using an ACQUITY UPLC BEH C18 column (1.7 μm, 2.1 × 100 mm; Waters Corporation, Milford, MA, USA) maintained at 40 °C. The autosampler temperature was maintained at 10 °C. Mobile phase A was 0.1% formic acid in water, and mobile phase B was 0.1% formic acid in acetonitrile/methanol (40:60, *v*/*v*). The injection volume was 5 μL. The gradient was programmed as follows: 10% B at 0 min, 40% B at 3 min, 95% B at 5 min, 100% B at 8–10 min, and 10% B at 10.5 min, followed by 3 min of re-equilibration. The flow rate was 0.25 mL/min from 0 to 5 min, increased to 0.60 mL/min from 8 to 10 min, and returned to 0.25 mL/min at 10.5 min.

Data were acquired separately in positive and negative ion modes using full-scan and data-dependent MS/MS acquisition. Full-scan data were collected at a resolution of 60,000 over an m/z range of 60–900, with an AGC target of 3 × 10^6^ and a maximum injection time of 100 ms. For MS/MS acquisition, the top 20 precursor ions were fragmented at a resolution of 15,000, with an AGC target of 2 × 10^5^, a maximum injection time of 25 ms, and stepped normalized collision energies of 20, 30, and 40. Raw LC–MS data were processed using the XCMS and Spectra R packages for peak detection, retention-time correction, alignment, and metabolite annotation. Metabolite features were annotated against the MoNA, GNPS, HMDB, MS-DIAL LipidBlast, and in-house databases. Metabolite intensities were log_2_-transformed and median-normalized. Metabolites with missing values in more than 30% of the samples were removed, and the remaining missing values were imputed using the K-nearest neighbors method. Orthogonal partial least squares discriminant analysis (OPLS-DA) was performed using the ropls package (version 1.36.0) in R. The model included one predictive and one orthogonal component with unit-variance scaling, and Q^2^ was estimated by seven-fold cross-validation. Model stability was further assessed by exact balanced-label permutation using all 252 possible assignments of five samples to each group. OPLS-DA was used as a descriptive multivariate analysis. Differential metabolite features were defined by VIP > 1, FDR < 0.05, and |log_2_ fold change| > 1. Functional annotation and pathway enrichment were performed using the HMDB and KEGG databases.

### 2.8. Exploratory Correlation Analysis

Sample-level pathway scores were calculated from the rumen epithelial transcriptomic data using ssGSEA with KEGG gene sets in the GSVA package (version 2.2.1) in R. Spearman rank correlations were calculated between differentially abundant microbial genera and differential VFAs, between differential VFAs and differential rumen epithelial morphological traits, and between differential VFAs and scores of differentially enriched metabolic and fatty acid-related pathways in the rumen epithelium. Correlations with |r| ≥ 0.70 and nominal *p* < 0.05 were highlighted for exploratory interpretation. FDR-adjusted *p* values and confidence intervals were also calculated for the correlation estimates. Correlation heatmaps were generated using the pheatmap package (version 1.0.12). Correlations between microbial genera and VFA concentrations and between VFA concentrations and epithelial morphology were based on 20 animals. Correlations between VFA concentrations and epithelial pathway scores were based on the 8 animals used for RNA sequencing.

### 2.9. Statistical Analysis

For conventional non-omics variables, normality and homogeneity of variance were assessed using the Shapiro–Wilk test and F test, respectively. Two-sided Student’s *t*-test was used when the data were normally distributed and variances were homogeneous; otherwise, the Wilcoxon rank-sum test was used. For rumen epithelial morphology, effects were reported as mean differences for Student’s *t*-tests or Hodges–Lehmann estimates for Wilcoxon rank-sum tests, with 95% confidence intervals. Statistical analyses were performed in R, and figures were generated using ggplot2 (version 4.0.3). Statistical significance was set at *p* < 0.05.

## 3. Results

### 3.1. Effects of Schizochytrium Oil on Body Weight

Initial and final body weights did not differ between the CK and SO groups (*p* > 0.05), whereas overall ADG tended to be higher in the SO group (0.33 vs. 0.29 kg/day, *p* = 0.06; Table 3).

### 3.2. Effects of Schizochytrium Oil on Rumen Epithelial Morphology

**Figure 1:**
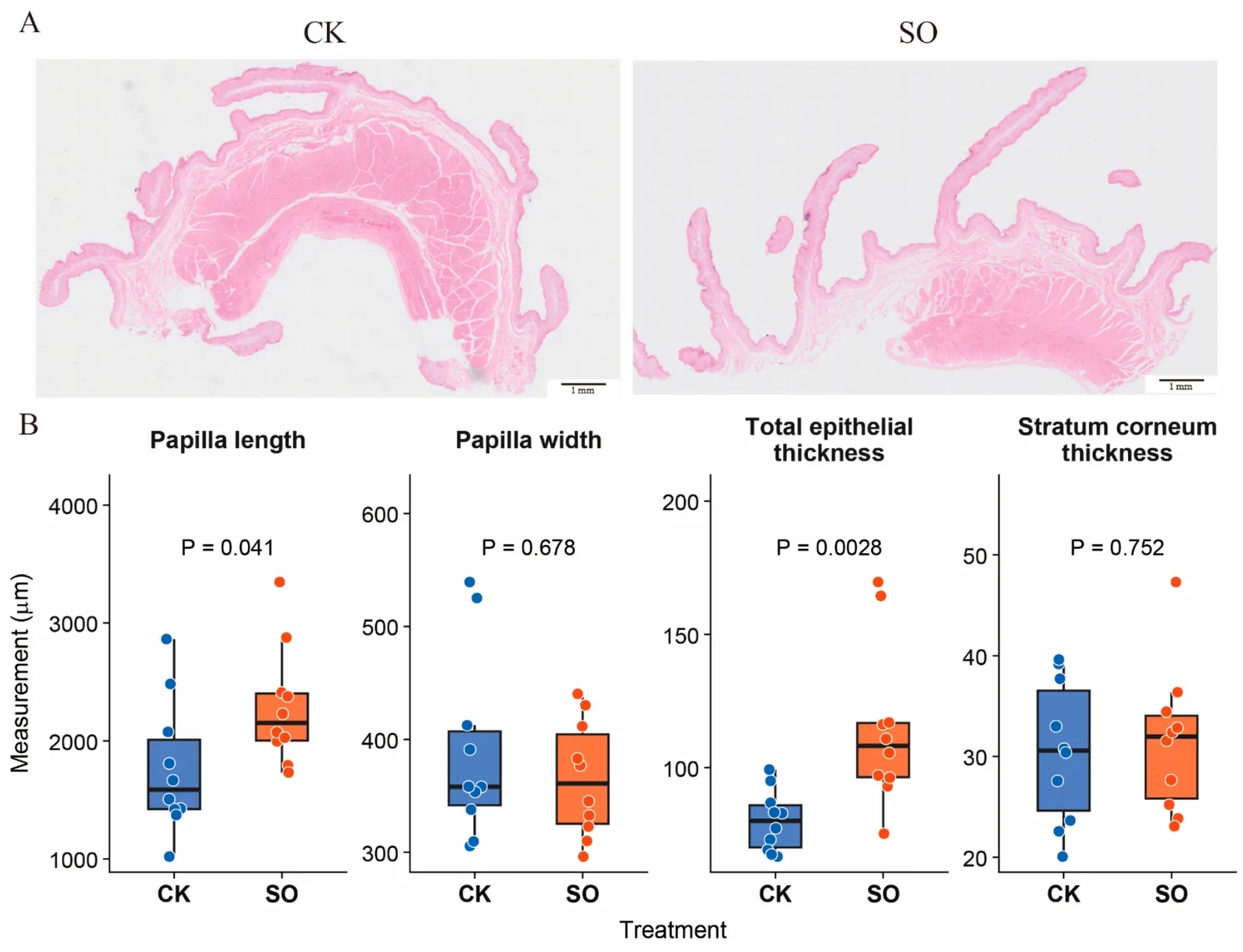
Effects of dietary *Schizochytrium* oil supplementation on rumen epithelial morphology in *Hu* lambs. (**A**) Representative hematoxylin and eosin (HE)-stained sections of rumen tissue from the CK and SO groups. Images were captured at 20× magnification. Scale bar = 1 mm. (**B**) Papilla length, papilla width, total epithelial thickness, and stratum corneum thickness of the rumen. Blue and orange represent the CK and SO groups, respectively. CK, control group; SO, *Schizochytrium* oil-supplemented group.

Compared with the CK group, the SO group had greater papilla length (means: 2286.49 vs. 1764.30 μm; mean difference = 522.20 μm, 95% CI: 22.48–1021.91, *p* = 0.041) and total epithelial thickness (means: 114.52 vs. 80.07 μm; Hodges–Lehmann estimate = 27.99 μm, 95% CI: 11.54–48.80, *p* = 0.003), whereas papilla width and stratum corneum thickness did not differ between groups (*p* > 0.05; Figure 1; Table S1).

### 3.3. Effects of Schizochytrium Oil on Ruminal VFA Concentrations at Slaughter

**Figure 2:**
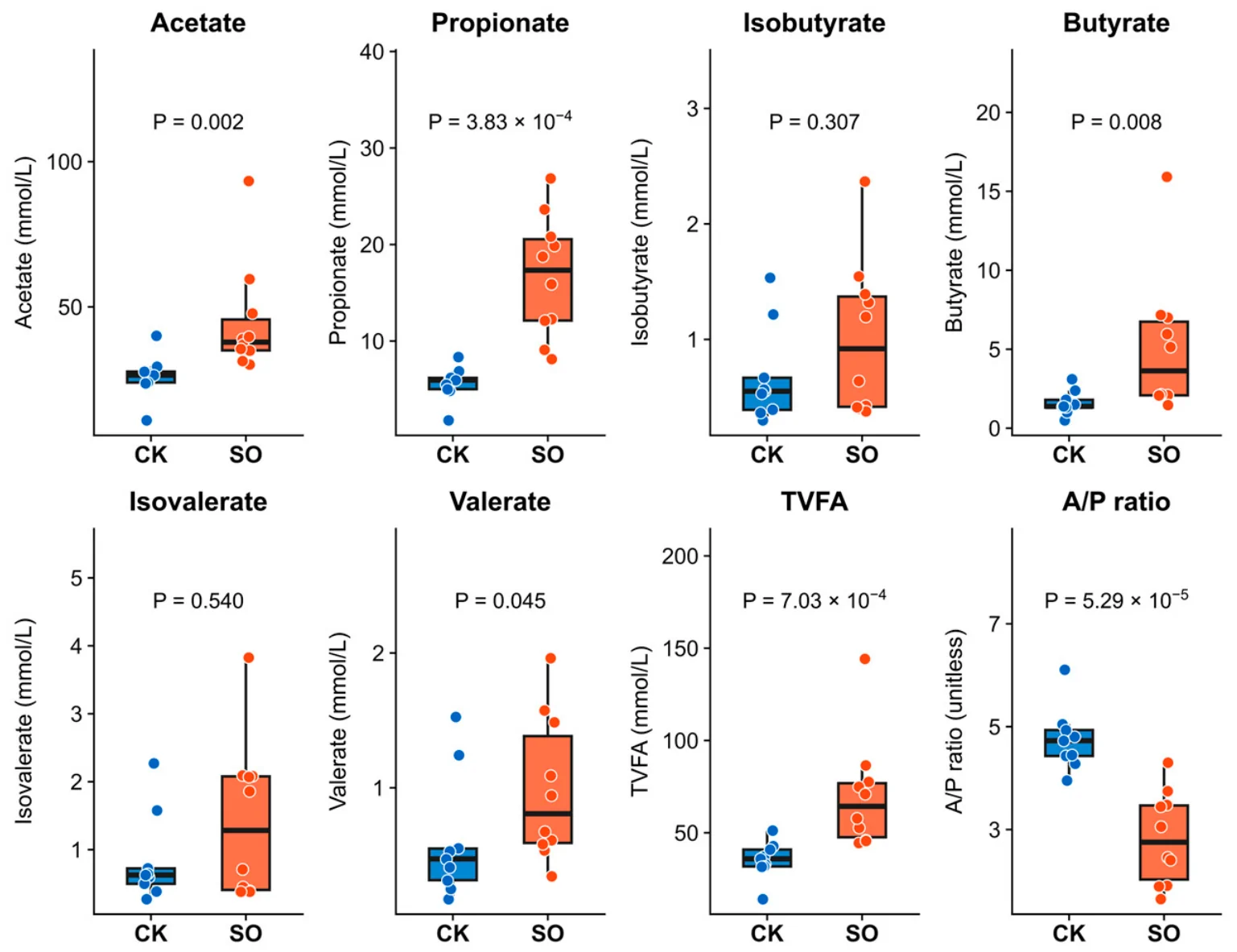
Ruminal volatile fatty acid concentrations at slaughter in *Hu* lambs fed the CK or SO diet. Rumen fluid was collected within 15 min after slaughter following a 16-h preslaughter fast. Blue and orange represent the CK and SO groups, respectively. CK, control group; SO, Schizochytrium oil group; TVFA, total volatile fatty acids; A/P, acetate-to-propionate ratio.

Compared with the CK group, the SO group had higher concentrations of acetate, propionate, butyrate, valerate, and total VFAs (*p* < 0.05) and a lower acetate/propionate ratio (*p* < 0.01; Figure 2). Isobutyrate and isovalerate concentrations did not differ between groups (*p* > 0.05).

### 3.4. Effects of Schizochytrium Oil on the Rumen Microbial Community

**Figure 3:**
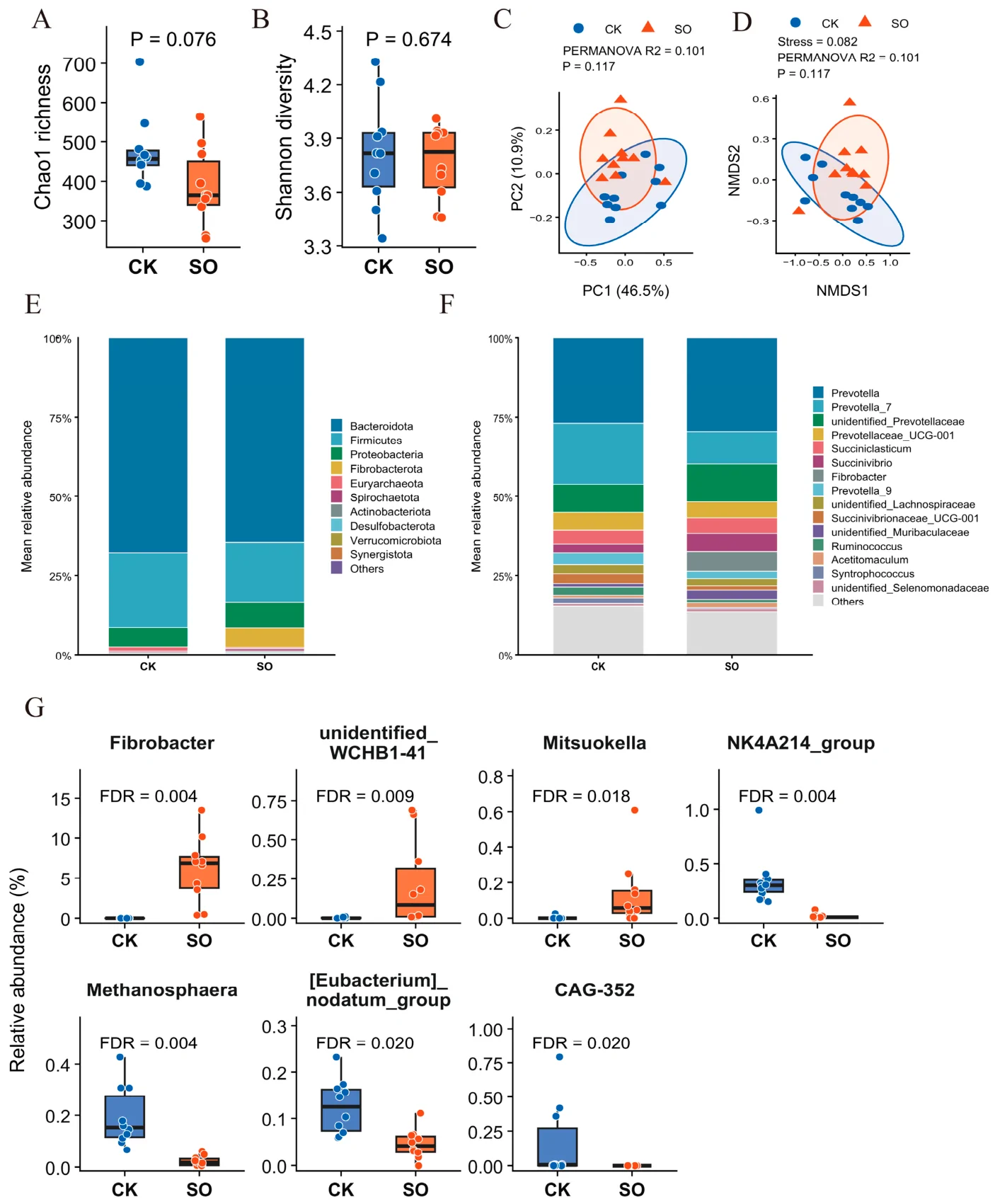
Effects of dietary *Schizochytrium* oil supplementation on rumen microbial diversity and community composition in Hu lambs. (**A**) Chao1 richness index. (**B**) Shannon diversity index. (**C**) Principal coordinate analysis (PCoA) based on Bray–Curtis distances. (**D**) Non-metric multidimensional scaling (NMDS) analysis based on Bray–Curtis distances. PERMANOVA results are shown in panels C and D. (**E**) Relative abundance of rumen microbiota at the phylum level. (**F**) Relative abundance of rumen microbiota at the genus level. (**G**) Relative abundance of differential genera between the CK and SO groups. FDR values are shown above each comparison. CK, control group. SO, *Schizochytrium* oil group.

A total of 52,045–131,354 sequences were obtained per sample, with a mean of 96,425 sequences. Good’s coverage ranged from 99.8886% to 99.9904% after rarefaction to the minimum sequencing depth of 52,045 sequences per sample, indicating high sequencing coverage (Table S2). The rarefaction curves are shown in Figure S1. A total of 2277 ASVs were detected, with 793 shared between the CK and SO groups. The CK and SO groups contained 873 and 611 group-specific ASVs, respectively (Figure S2). The Chao1 and Shannon indices did not differ significantly between the CK and SO groups (*p* > 0.05; Figure 3A,B). PCoA showed that the first and second principal coordinates explained 46.5% and 10.9%, respectively (Figure 3C). PERMANOVA detected no significant difference in overall microbial community structure between the CK and SO groups (R^2^ = 0.101, *p* > 0.05; Figure 3C). The NMDS stress value was 0.082 (Figure 3D), and PERMDISP showed no significant difference in multivariate dispersion between the two groups (*p* > 0.05).

At the phylum level, Bacteroidota, Firmicutes, and Proteobacteria were the dominant phyla in both the CK and SO groups (Figure 3E). At the genus level, *Prevotella*, *Prevotella_7*, *unidentified_Prevotellaceae*, *Prevotellaceae_UCG-001*, and *Succiniclasticum* were the predominant genera in both groups (Figure 3F). A total of seven differential genera were identified, with higher relative abundances of *Fibrobacter*, *unidentified_WCHB1-41*, and *Mitsuokella* and lower relative abundances of *NK4A214_group*, *Methanosphaera*, *[Eubacterium]_nodatum_group*, and *CAG-352* in the SO group (FDR < 0.05; Figure 3G).

### 3.5. Effects of Schizochytrium Oil on Rumen Epithelial Transcriptomic Profiles

**Figure 4:**
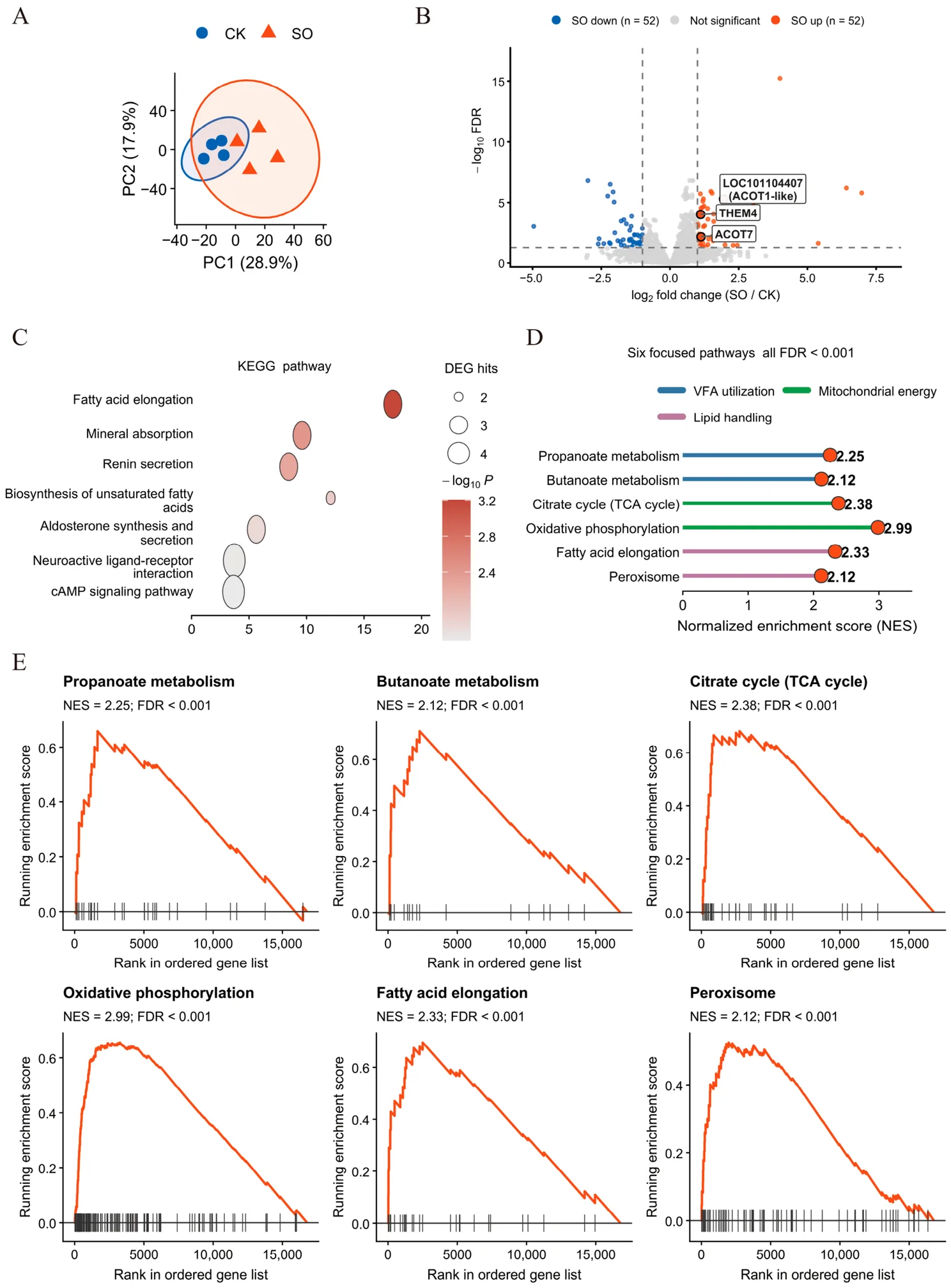
Effects of *Schizochytrium* oil on Rumen Transcriptomic Profiles. (**A**) Principal component analysis (PCA) of rumen epithelial transcriptomic profiles. (**B**) Volcano plot of differentially expressed genes (DEGs) between the SO and CK groups. Red dots indicate upregulated genes, blue dots indicate downregulated genes, and gray dots indicate genes that did not meet the significance threshold. (**C**) KEGG pathway enrichment analysis of DEGs. (**D**) Normalized enrichment scores (NES) of selected metabolic and fatty acid-related pathways identified by gene set enrichment analysis (GSEA). (**E**) GSEA plots of propanoate metabolism, butanoate metabolism, citrate cycle (TCA cycle), oxidative phosphorylation, fatty acid elongation, and peroxisome. CK, control group. SO, *Schizochytrium* oil group.

RNA sequencing generated 47.07-48.14 million clean reads per sample, with Q30 values of 96.85–97.41% and 97.05–98.15% of reads mapped to the sheep reference genome (Table S3). In the PCA, PC1 and PC2 explained 28.9% and 17.9% of the total variance, respectively (Figure 4A). A total of 104 DEGs were identified, including 52 upregulated and 52 downregulated genes in the SO group compared with the CK group (Figure 4B, Table S4). KEGG pathway enrichment analysis identified seven pathways with *p* < 0.05, including fatty acid elongation, mineral absorption, renin secretion, biosynthesis of unsaturated fatty acids, aldosterone synthesis and secretion, neuroactive ligand–receptor interaction, and the cAMP signaling pathway. However, none of these pathways had FDR < 0.05 (Figure 4C; Table S5). GSEA further showed that propanoate metabolism, butanoate metabolism, the citrate cycle (TCA cycle), oxidative phosphorylation, fatty acid elongation, and peroxisome were enriched in the SO group, with all six pathways having FDR < 0.01 (Figure 4D,E; Table S6). Among these six pathways, three DEGs, *LOC101104407 (ACOT1-like)*, *ACOT7*, and *THEM4*, were significantly upregulated in the SO group (FDR < 0.05, Figure S3).

### 3.6. Effects of Schizochytrium Oil on the Serum Metabolomic Profile

**Figure 5:**
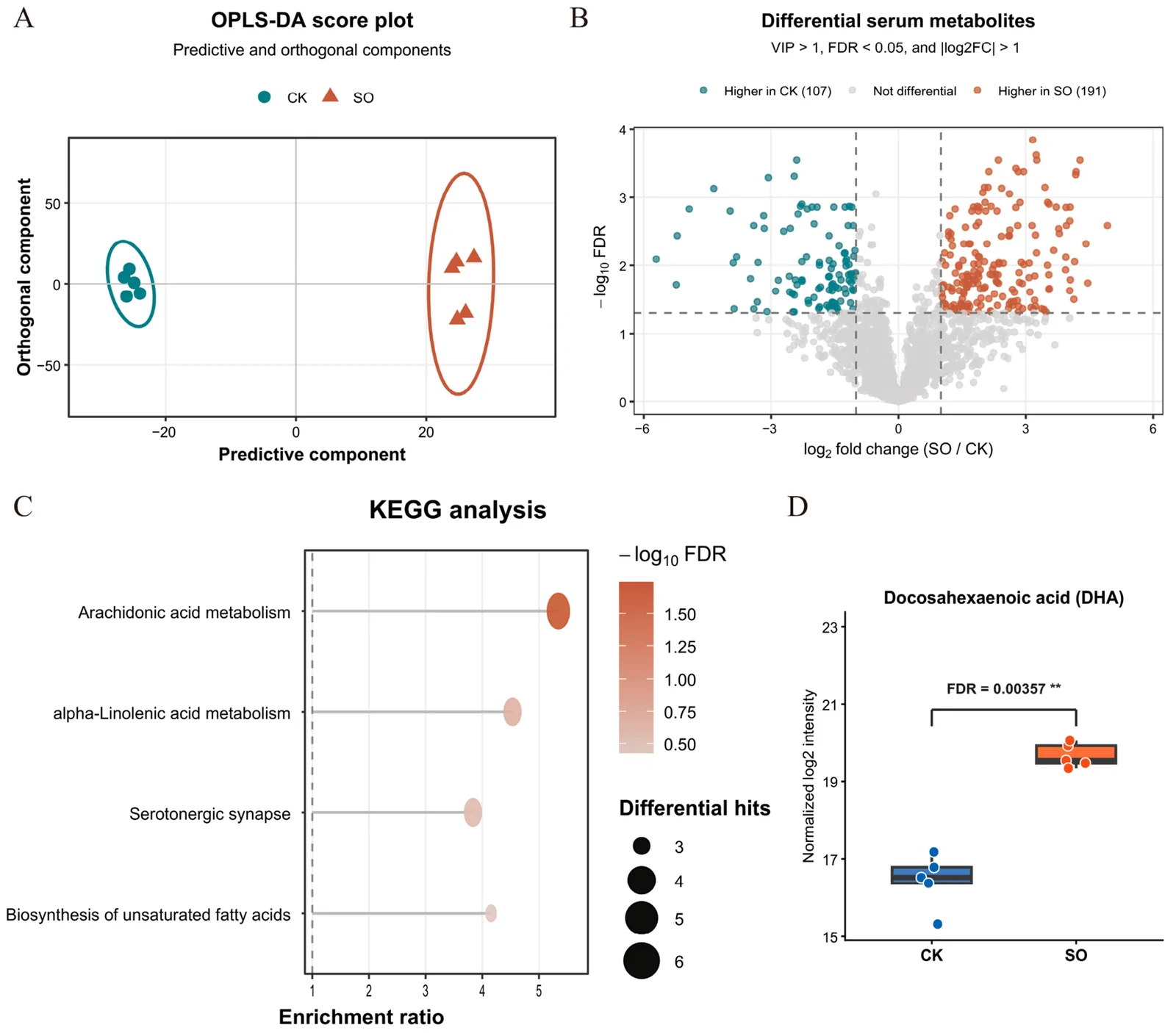
Effects of *Schizochytrium* oil on the serum metabolomic profile of *Hu* lambs. (**A**) OPLS-DA score plot of serum metabolomic profiles in the CK and SO groups. (**B**) Volcano plot of differential serum metabolites. Differential metabolites were identified using VIP > 1, FDR < 0.05, and |log_2_FC| > 1. (**C**) KEGG pathway analysis of differential serum metabolites. Dot size represents the number of differential metabolites, and color represents −log_10_ FDR. (**D**) Serum docosahexaenoic acid (DHA) level in the CK and SO groups. Statistical significance was defined as FDR < 0.05. CK, control group. SO, *Schizochytrium* oil group. ** FDR < 0.01.

The OPLS-DA score plot showed an apparent separation between the CK and SO groups (Figure 5A). The model had R^2^X(cum), R^2^Y(cum), and Q^2^(cum) values of 0.425, 0.998, and 0.890, respectively (Figure S4A). Exhaustive balanced-label permutation across all 252 possible group assignments gave an empirical *p* = 0.0079 for both R^2^Y(cum) and Q^2^(cum) (Figure S4B). A total of 298 differential metabolite features were identified, including 191 higher and 107 lower in the SO group (Figure 5B; Table S7). KEGG analysis showed enrichment in arachidonic acid metabolism, alpha-linolenic acid metabolism, serotonergic synapse, and biosynthesis of unsaturated fatty acids (Figure 5C). The serum metabolite feature annotated as DHA was significantly higher in the SO group than in the CK group (FDR < 0.01; Figure 5D). DHA-containing serum lipids are shown in Figure S5, and the SO group showed higher levels of most DHA-containing lipid species than the CK group, including PC(16:1/22:6), PC(18:1/22:6), and PC(15:1/22:6). At the lipid subclass level, DHA-containing PC, PE, LPE, and PA/LPA were significantly higher in the SO group than in the CK group (FDR < 0.05).

### 3.7. Associations Among Differential Rumen Genera, VFA Profiles, and Epithelial Metabolic Pathways

**Figure 6:**
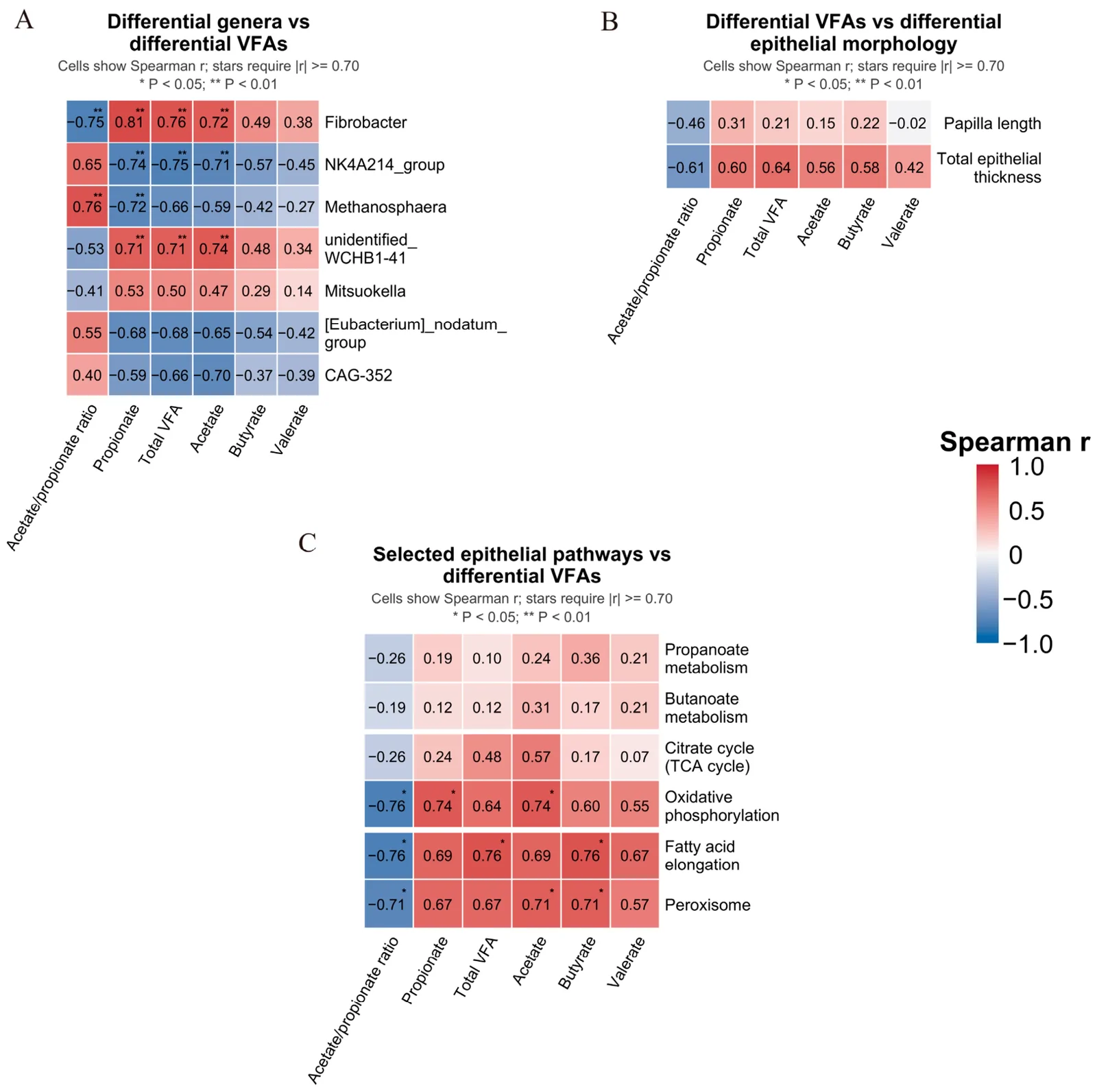
Spearman correlations among differential rumen genera, differential VFAs, rumen epithelial morphology, and selected epithelial pathways in Hu lambs. (**A**) Correlations between differential genera and differential VFA concentrations. (**B**) Correlations between differential VFA concentrations and differential rumen epithelial morphological traits. (**C**) Correlations between differential VFA concentrations and selected metabolic and fatty acid-related pathways in the rumen epithelium. Values in the cells represent Spearman correlation coefficients. Correlations with |r| ≥ 0.70 are highlighted. *p* values are nominal, and the correlation analyses are exploratory. * *p* < 0.05, ** *p* < 0.01. VFA, volatile fatty acid; A/P, acetate-to-propionate ratio. Sample sizes were *n* = 20 (10 per group) for panels (**A**,**B**) and *n* = 8 (4 per group) for panel (**C**). *p* values were not adjusted for multiple comparisons.

Spearman correlation analysis showed that Fibrobacter was negatively correlated with the acetate/propionate ratio (r = −0.75, *p* < 0.01) and positively correlated with propionate (r = 0.81, *p* < 0.01), total VFAs (r = 0.76, *p* < 0.01), and acetate (r = 0.72, *p* < 0.01; Figure 6A). NK4A214_group was negatively correlated with propionate (r = −0.74, *p* < 0.01), total VFAs (r = −0.75, *p* < 0.01), and acetate (r = −0.71, *p* < 0.01). Methanosphaera was positively correlated with the acetate/propionate ratio (r = 0.76, *p* < 0.01) and negatively correlated with propionate (r = −0.72, *p* < 0.01). unidentified_WCHB1-41 was positively correlated with propionate (r = 0.71, *p* < 0.01), total VFAs (r = 0.71, *p* < 0.01), and acetate (r = 0.74, *p* < 0.01). No strong correlations (|r| ≥ 0.70) were observed between the differential VFA concentrations and papilla length or total epithelial thickness (Figure 6B). Among the selected epithelial pathways, oxidative phosphorylation was negatively correlated with the acetate/propionate ratio (r = −0.76, *p* < 0.05) and positively correlated with propionate (r = 0.74, *p* < 0.05) and acetate (r = 0.74, *p* < 0.05; Figure 6C). Fatty acid elongation was negatively correlated with the acetate/propionate ratio (r = −0.76, *p* < 0.05) and positively correlated with total VFAs and butyrate (both r = 0.76, *p* < 0.05). Peroxisome was negatively correlated with the acetate/propionate ratio (r = −0.71, *p* < 0.05) and positively correlated with acetate and butyrate (both r = 0.71, *p* < 0.05). Complete correlation results, including FDR-adjusted *p* values and confidence intervals, are provided in Table S8.

## 4. Discussion

In the present study, SO supplementation did not significantly affect the Chao1 or Shannon index of the rumen microbiota, and PERMANOVA also showed no significant difference in the overall microbial community between the CK and SO groups. Bacteroidota, Firmicutes, and Proteobacteria were the dominant phyla in both groups, while *Prevotella* and several *Prevotellaceae*-related genera were predominant at the genus level. Thus, SO supplementation did not result in a broad change in the overall rumen microbial community. Similar results have been reported in goats receiving *Schizochytrium*, in which estimated DHA intakes of 4.16–8.44 g/animal/day did not significantly affect Shannon diversity, and Bacteroidetes, Firmicutes, and Proteobacteria remained the dominant phyla [17].

Among the differential genera, the relative abundance of *Methanosphaera* was significantly lower in the SO group, consistent with a previous goat study in which the estimated daily DHA intake ranged from 4.16 to 8.44 g/goat/day [18]. *Methanosphaera* is an H_2_-dependent methylotrophic methanogen that mainly uses H_2_ to reduce methanol to CH_4_. Its growth is closely related to the availability of H_2_ and methanol in the rumen [19]. The decrease in *Methanosphaera* may be related to the sensitivity of methanogens to long-chain PUFAs. Previous culture studies have shown that long-chain PUFAs can damage the cell membrane of methanogens and inhibit their growth and methanogenic activity [20,21]. Interestingly, the relative abundance of *Fibrobacter* was significantly higher in the SO group, which differs from a previous goat study reporting lower F. succinogenes abundance at estimated DHA intakes of 4.16–8.44 g/goat/day after *Schizochytrium* supplementation [22]. *Fibrobacter* is an important fibrolytic bacterium in the rumen and has a strong ability to degrade cellulose [23]. One possible explanation may be related to the lower palm oil level in the SO diet. Previous work in goats showed that palm oil reduced the abundance of *F. succinogenes* [24]. The lower palm oil level in the SO diet may therefore have contributed to the higher relative abundance of *Fibrobacter*. However, because SO partially replaced palm oil, the effects of SO addition and reduced palm oil cannot be separated. In addition, the present study measured microbial relative abundance by 16S rRNA sequencing rather than absolute abundance. Therefore, the higher relative abundance of Fibrobacter does not necessarily indicate an increase in its absolute abundance [25]. The relative abundance of *NK4A214_group* was significantly lower in the SO group. Previous studies suggest that *NK4A214_group* is involved in the degradation of fiber and resistant starch in the rumen [26,27]. Its abundance has also been associated with ruminal VFA concentrations, including propionate and butyrate [26]. Notably, the relative abundance of *NK4A214_group* was lower, whereas that of Fibrobacter was higher in the SO group, suggesting that SO supplementation may have changed the relative distribution of different functional microbial groups. Previous studies on PUFA supplementation have also reported different responses among bacteria involved in fiber and carbohydrate utilization [11]. These findings further suggest that the effects of SO on the rumen microbiota may be highly taxon specific.

In the present study, the SO group had higher ruminal concentrations of acetate, propionate, butyrate, valerate, and total VFAs at slaughter than the CK group. Propionate is one of the major gluconeogenic precursors in ruminants and provides an important source of glucose for maintenance and growth [28]. Previous studies have estimated that propionate provides approximately 60–74% of the carbon used for hepatic glucose production in ruminants [28]. Higher propionate concentrations have often been associated with improved feed efficiency [29], and overall ADG tended to be higher in the SO group in the present study. The differences in VFA concentrations were also associated with several differential rumen genera. *Fibrobacter* was positively correlated with propionate and total VFAs, whereas *unidentified_WCHB1-41* was positively correlated with propionate, total VFAs, and acetate. The major metabolic products of *Fibrobacter* include succinate, acetate, and formate [23], and succinate is an important intermediate in propionate metabolism. Previous studies have also reported positive correlations between *Fibrobacter* and ruminal VFA concentrations [30], while *unidentified_WCHB1-41* has been positively correlated with total VFAs in cattle [31]. In contrast, *Methanosphaera* was negatively correlated with propionate, consistent with previous observations in sheep [32]. Overall, SO supplementation significantly altered ruminal VFA concentrations at slaughter and was accompanied by differences in the relative abundance of several rumen genera.

SO supplementation also affected rumen epithelial morphology and related transcriptional responses. In the present study, papilla length and total rumen epithelial thickness were significantly increased in the SO group, together with higher ruminal propionate and butyrate concentrations. Previous studies have shown that increased rumen papilla growth can be accompanied by enhanced short-chain fatty acid absorption [33]. Butyrate is also an important regulator of rumen epithelial growth. Previous intervention studies have shown that butyrate can promote rumen epithelial growth by regulating processes such as the cell cycle and protein synthesis [12,34]. The importance of VFAs and dietary composition for rumen epithelial development has also been discussed in young ruminants [35]. Butyrate and butanoate metabolism have also been associated with oxidative phosphorylation and rumen papilla development [36]. However, none of the correlations between VFA concentrations and papilla length or total epithelial thickness reached the prespecified threshold for a strong correlation. This may be because the two measurements reflect changes over different periods of time. Rumen epithelial morphology develops gradually over the feeding period, whereas VFA concentrations were measured only once at slaughter after fasting. Therefore, the terminal VFA concentrations provide limited evidence for explaining the morphological differences that developed over the feeding period.

At the transcriptional level, pathways related to propanoate metabolism, butanoate metabolism, the TCA cycle, and oxidative phosphorylation showed transcriptional enrichment in the SO group, indicating changes in the expression of genes related to VFA utilization and energy metabolism in the rumen epithelium. Propionate can be converted to succinyl-CoA and enter the TCA cycle [37], providing a metabolic basis for its association with transcriptional changes related to the TCA cycle and oxidative phosphorylation. Previous studies in yaks also found positive correlations between ruminal propionate and VFA transporter expression in the rumen epithelium, including *MCT1* and *PAT1* [13], suggesting that changes in propionate concentration may be accompanied by VFA transport-related responses in the rumen epithelium. In the present study, propionate was strongly positively correlated with the oxidative phosphorylation pathway score, and total VFAs was also positively correlated with this pathway, consistent with the transcriptomic results described above. SO supplementation also induced transcriptional changes related to lipid metabolism in the rumen epithelium. The fatty acid elongation pathway was significantly enriched in the SO group, with increased expression of *ACOT7* and *THEM4*. *ACOT7* has high activity toward long-chain PUFA-CoA, including DHA-CoA, whereas *THEM4* is involved in the regulation of intracellular acyl-CoA and free CoA balance [38,39,40]. Therefore, the increased expression of *ACOT7* and *THEM4* may reflect a transcriptional adaptation of the rumen epithelium to altered lipid exposure after SO supplementation.

Serum metabolites can reflect the nutrient substrates available to the host after dietary treatment [41,42]. In the present study, the serum feature annotated as DHA was significantly higher in the SO group, which had an estimated average DHA intake of 6.16 g/lamb/day. This is consistent with a previous goat study showing increased plasma DHA at estimated DHA intakes of 4.16–8.44 g/goat/day after *Schizochytrium* supplementation [43]. Importantly, several glycerophospholipids containing 22:6 acyl chains were also higher in the SO group, including PC(16:1/22:6), PC(18:1/22:6), and PC(15:1/22:6). The lipid carrier form of DHA can influence its uptake and utilization in some tissues [44,45], suggesting that the increase in DHA-containing serum lipids may be biologically relevant. In addition, pathways related to α-linolenic acid and unsaturated fatty acid metabolism were enriched in the SO group. These changes further show that SO supplementation altered the circulating n-3 lipid profile.

This study has several main limitations. First, each dietary treatment was applied to a single pen, and feed intake was recorded at the pen level rather than for individual lambs. Second, the relatively small RNA-seq and serum metabolomics sample sizes may limit statistical power. Third, ruminal pH was not measured, which limits the characterization of the ruminal environment. Finally, SO replaced part of the palm oil rather than being added under an identical lipid background, so the observed differences may reflect the addition of SO, the reduction in palm oil, or both.

## 5. Conclusions

Under the conditions of this study, partial replacement of palm oil with 1% SO increased the estimated average DHA intake from 2.24 to 6.16 g/lamb/day and was associated with changes in the relative abundance of selected rumen genera, including higher *Fibrobacter* and lower *Methanosphaera*. The SO group also had higher acetate, propionate, butyrate, valerate, and total VFA concentrations and a lower acetate/propionate ratio at slaughter. In addition, papilla length and total epithelial thickness were greater in the SO group, together with transcriptional enrichment of pathways related to propanoate metabolism, butanoate metabolism, the TCA cycle, and oxidative phosphorylation. Serum DHA and several DHA-containing lipids were higher in the SO group. Overall, partial replacement of palm oil with 1% SO in a medium-fiber diet was accompanied by changes in rumen microbiota, ruminal VFA concentrations at slaughter, the rumen epithelial transcriptome, and the serum metabolome in *Hu* lambs. Further studies with the larger omics sample sizes are needed to confirm these integrated associations.

## Figures and Tables

**Table 1:** Ingredient composition and analyzed nutrient levels of the experimental diets.

Items	CK	SO
Ingredients (%)		
Corn stover	24	24
Corn husks	8	8
Cracked corn	26	26
Soybean meal	5	5
Cottonseed meal	6	6
Corn germ meal	20	20
Palm oil	4	3
*Schizochytrium* oil	0	1
Puffed urea	1	1
Molasses	3	3
NaCl	0.5	0.5
Limestone	1.2	1.2
Sodium bicarbonate	0.8	0.8
Premix	0.5	0.5
Total	100	100
Nutrients (%)		
DM	92.26	92.11
Ash content	6.94	6.91
EE	5.58	5.45
NDF	31.43	31.76
ADF	15.60	14.88
CP	15.42	15.31

Note: CK, control diet containing 4% palm oil; SO, diet containing 3% palm oil and 1% Schizochytrium oil; DM, dry matter; EE, ether extract; NDF, neutral detergent fiber; ADF, acid detergent fiber; CP, crude protein. The premix provided vitamins and minerals. Each kilogram of diet dry matter contained 2500 IU vitamin A, 23 IU vitamin E, 0.3 mg selenium, 70 mg iron, 41 mg zinc, and 8 mg copper.

**Table 2:** Fatty acid composition of the CK and SO diets.

Fatty Acids (%)	CK	SO
C12:0	0.30	0.26
C14:0	0.73	0.75
C16:0	50.67	48.32
C16:1	0.15	0.15
C17:0	0.12	0.13
C18:0	8.27	6.97
C18:1n9t	8.95	7.93
C18:1n9c	0.00	0.78
C18:2n6t	24.96	24.68
C20:0	0.29	0.31
C18:3n6	0.04	0.06
C18:3n3	2.63	2.48
C20:2	0.07	0.09
C22:0	0.21	0.21
C20:3n6	0.04	0.07
C20:3n3	0.12	0.17
C22:2	0.13	0.21
C24:0	0.12	0.14
C20:5n3	0.03	0.09
C22:6n3	2.18	6.20
ΣSFA	60.71	57.09
ΣMUFA	9.10	8.86
ΣPUFA	30.19	34.04
Σn-3 PUFA	4.95	8.93
Σn-6 PUFA	25.04	24.81
Σn-3/n-6 PUFA	0.20	0.36

**Table 3:** Effects of dietary SO on body weight and average daily gain of *Hu* lambs.

Item	CK	SO	SEM	*p*-Value
Initial body weight (kg)	21.93	21.75	0.98	0.86
Final body weight (kg)	46.48	49.27	2.12	0.23
Overall ADG (kg/day)	0.29	0.33	0.02	0.06

Note: CK, control group; SO, *Schizochytrium* oil group; ADG, average daily gain.

## Data Availability

The original data presented in the study are openly available in CNGB (https://db.cngb.org/) at CNP0010019 and CNP0010018.

## Supplementary Materials

The following supporting information can be downloaded at: https://www.mdpi.com/article/10.3390/microorganisms14102268/s1, Figure S1: Rarefaction curves of rumen microbial ASVs in the CK and SO groups; Figure S2: Shared and group-specific rumen microbial ASVs in the CK and SO groups; Figure S3: Differentially expressed genes within the six selected GSEA pathways in the rumen epithelium; Figure S4: OPLS-DA model parameters and exhaustive balanced-label permutation results for the serum metabolomic data; Figure S5: DHA- and 22:6-containing serum lipid profiles in Hu lambs; Table S1: Effect estimates and 95% confidence intervals for rumen epithelial morphology; Table S2: Sequencing depth and Good’s coverage of rumen microbiota samples; Table S3: Summary of rumen epithelial RNA-seq data quality; Table S4: Differentially expressed genes in the rumen epithelium; Table S5: KEGG enrichment analysis of rumen epithelial DEGs; Table S6: Complete GSEA results of rumen epithelial transcriptomic data; Table S7: Differential serum metabolite features between the CK and SO groups, including annotation information; Table S8: Complete Spearman correlation results, including FDR-adjusted *p* values and confidence intervals.

## Abbreviations

The following abbreviations are used in this manuscript:

CK	Control group
SO	Schizochytrium oil
VFA	Volatile fatty acid
A/P	Acetate-to-propionate ratio
PCoA	Principal coordinate analysis
NMDS	Non-metric multidimensional scaling
PERMANOVA	Permutational multivariate analysis of variance
DEG	Differentially expressed gene
GSEA	Gene set enrichment analysis
OPLS-DA	Orthogonal partial least squares discriminant analysis
