# Supplementary material for: Effects of Partial Replacement of Palm Oil with Schizochytrium Oil on Rumen Microbiota, Ruminal Volatile Fatty Acid Concentrations, Rumen Epithelial Transcriptome, and Serum Metabolome in Hu Lambs Fed a Medium-Fiber Diet
Source: Microorganisms. 2026 Oct 6;14(10):2268. doi: 10.3390/microorganisms14102268 (PMC13707486; doi:10.3390/microorganisms14102268)
Supplement: Supplementary file 1 [file microorganisms-14-02268-s001.zip › Supplementary Figures.pdf]

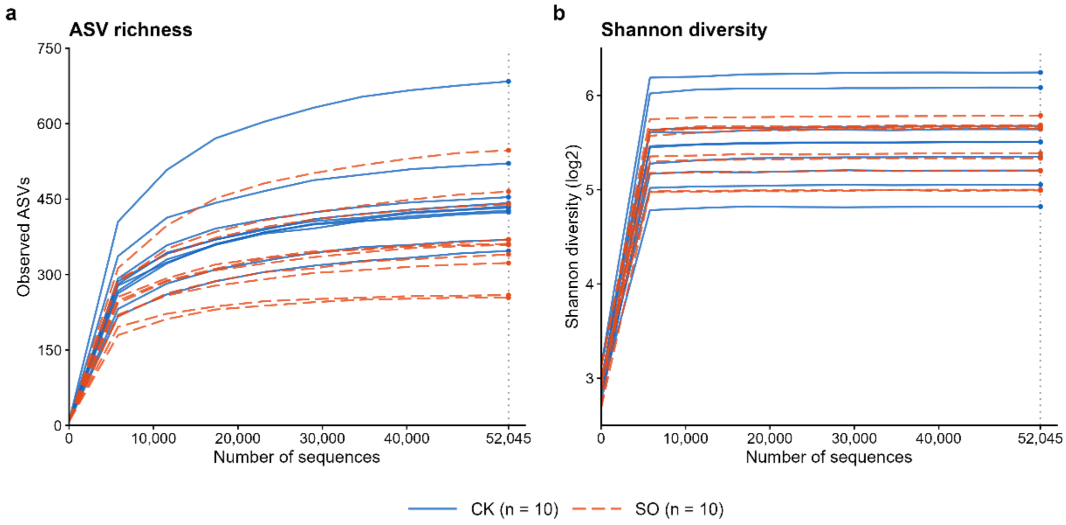

Figure S1. Rarefaction curves of rumen microbial communities in the CK and SO groups. (A) ASV richness. (B) Shannon diversity. Each curve represents one rumen sample. Blue solid lines represent the CK group (n = 10), and orange dashed lines represent the SO group (n = 10). The vertical dotted line indicates the standardized sequencing depth of 52,045 sequences per sample used for alpha-diversity analysis.

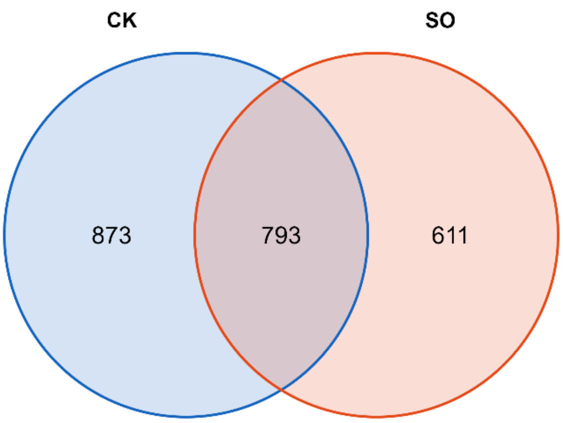

Figure S2. Shared and group-specific rumen microbial ASVs in the CK and SO groups. The Venn diagram shows 873 amplicon sequence variants (ASVs) detected only in the CK group, 611 detected only in the SO group, and 793 shared between both groups. These counts are presented descriptively and were not used to infer differences in microbial richness. CK, control group; SO, Schizochytrium oil group.

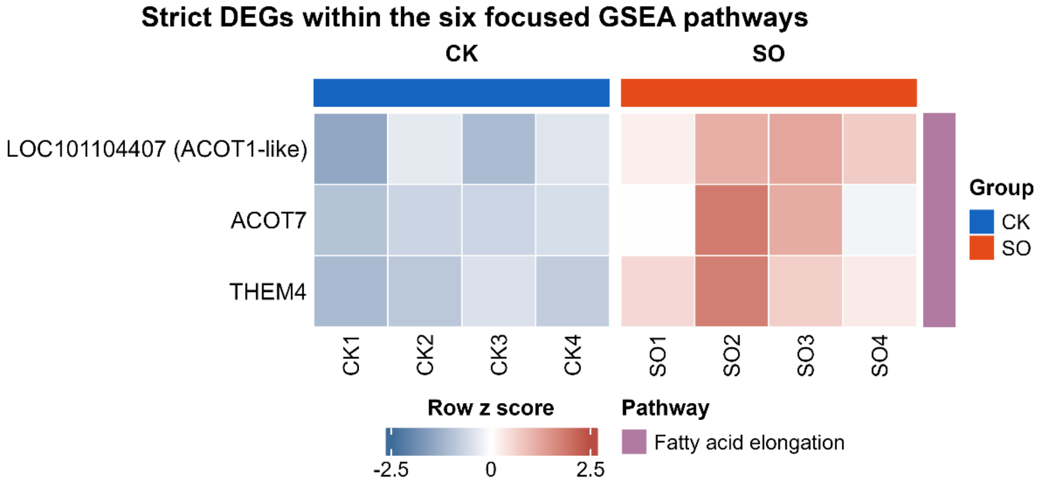

Figure S3. Differentially expressed genes within the six selected metabolic and fatty acid-related GSEA pathways in the rumen epithelium of Hu lambs fed the CK or SO diets. Heatmap shows LOC101104407 (ACOT1-like), ACOT7, and THEM4, which were significantly upregulated in the SO group (FDR < 0.05) and were involved in fatty acid elongation. CK, control group. SO, Schizochytrium oil group

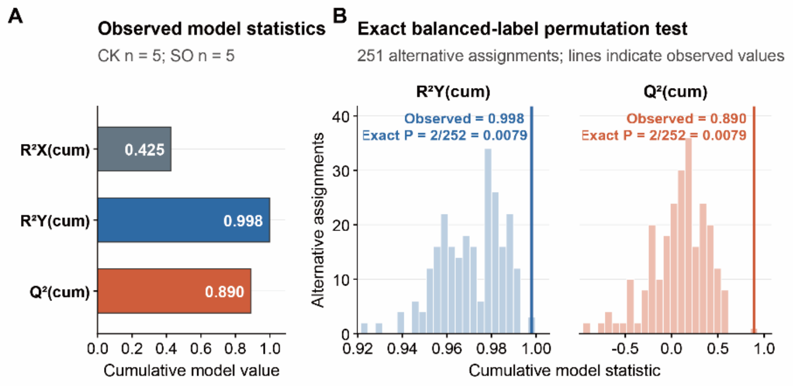

Figure S4. OPLS-DA model performance and exact balanced-label permutation analysis of serum metabolomic data. (A) The cumulative R^2X, R^2Y, and Q^2 values were 0.425, 0.998, and 0.890, respectively. (B) Distribution of R^2Y and Q^2 values from the 251 alternative balanced label assignments. Red lines indicate the observed values. Exact empirical P values were 0.0079 for both R^2Y and Q^2.

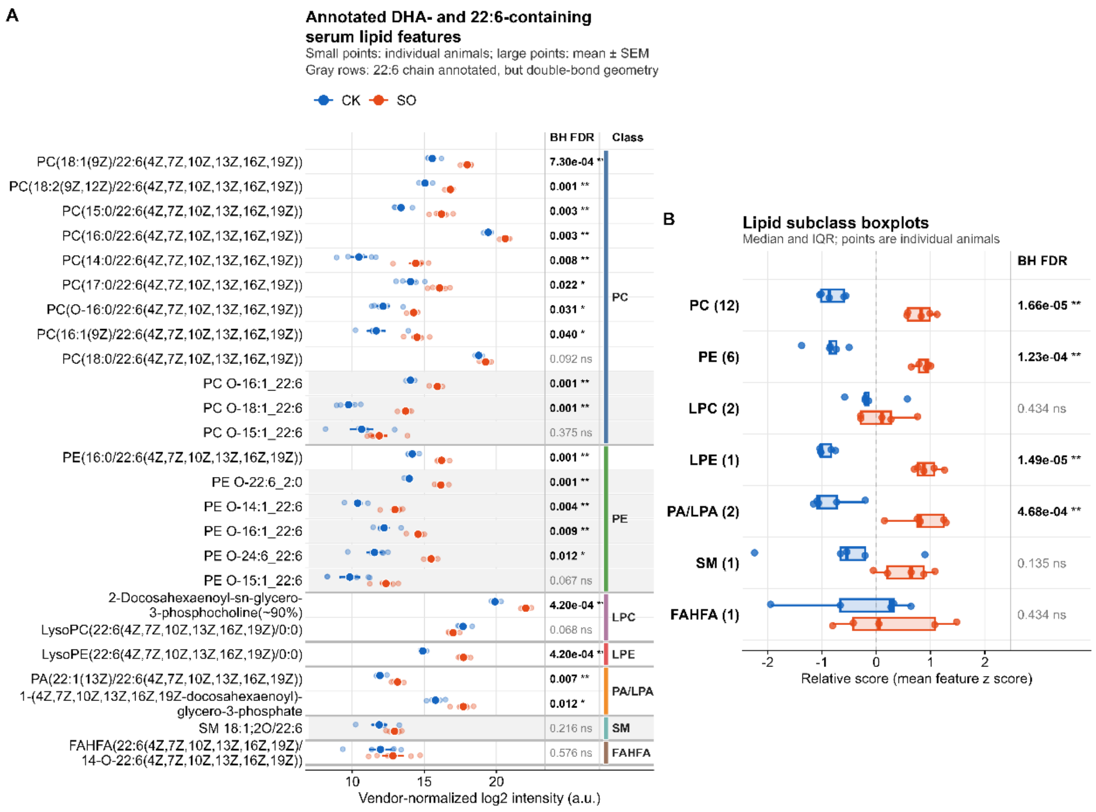

Figure S5. Effects of Schizochytrium Oil on DHA-Containing Serum Lipid Profiles in Hu Lambs (A) Relative levels of individual DHA- containing lipid species. (B) Relative levels of DHA-containing lipid subclasses, including PC, PE, LPC, LPE, PA/LPA, SM, and FAHFA. Statistical significance was defined as FDR < 0.05. CK, control group; SO, Schizochytrium oil group. PC, phosphatidylcholine; PE, phosphatidylethanolamine; LPC, lysophosphatidylcholine; LPE, lysophosphatidylethanolamine; PA, phosphatidic acid; LPA, lysophosphatidic acid; SM, sphingomyelin; FAHFA, fatty acid ester of hydroxy fatty acid.
